# Developing Protein-Based Nanoparticles as Versatile Delivery Systems for Cancer Therapy and Imaging

**DOI:** 10.3390/nano9091329

**Published:** 2019-09-16

**Authors:** Febrina Sandra, Nisar Ul Khaliq, Anwar Sunna, Andrew Care

**Affiliations:** 1 Department of Molecular Sciences, Macquarie University, Sydney 2109, Australia; febrina.sandra@hdr.mq.edu.au (F.S.); anwar.sunna@mq.edu.au (A.S.); 2 College of Pharmacy, Korea University, 2511 Sejong-ro, Sejong 30019, Korea; nisul151@korea.ac.kr; 3 Australian Research Council Centre of Excellence for Nanoscale BioPhotonics, Macquarie University, Sydney, NSW 2109, Australia

**Keywords:** drug delivery, nanomedicine, cancer therapy, cancer imaging, virus-like particles, protein-based nanoparticles, synthetic biology

## Abstract

In recent years, it has become apparent that cancer nanomedicine’s reliance on synthetic nanoparticles as drug delivery systems has resulted in limited clinical outcomes. This is mostly due to a poor understanding of their “bio–nano” interactions. Protein-based nanoparticles (PNPs) are rapidly emerging as versatile vehicles for the delivery of therapeutic and diagnostic agents, offering a potential alternative to synthetic nanoparticles. PNPs are abundant in nature, genetically and chemically modifiable, monodisperse, biocompatible, and biodegradable. To harness their full clinical potential, it is important for PNPs to be accurately designed and engineered. In this review, we outline the recent advancements and applications of PNPs in cancer nanomedicine. We also discuss the future directions for PNP research and what challenges must be overcome to ensure their translation into the clinic.

## 1. Introduction

Cancer nanomedicine is defined as implementing nanotechnology for the diagnosis and treatment of cancer [1,2]. It predominantly relies upon nanoparticles (NPs), which are inorganic and/or organic materials with nanoscale dimensions (1–100 nm), to act as delivery vehicles for imaging or therapeutic agents [3]. These nanoparticle-based drug delivery systems (NDDSs) have the potential to overcome many of the challenges associated with drug delivery in conventional cancer therapies, such as: enabling specific drug targeting; enhancing drug physicochemical properties (e.g., stability and solubility); improving drug pharmacokinetics and biodistribution; lowering drug toxicity; assisting cellular uptake; and facilitating uniform drug dosing—all of which can ultimately enhance clinical outcomes for patients. In addition, many NPs have useful optical properties and can also be loaded with optically-active agents (e.g., fluorophores), allowing NDDSs to provide better contrast enhancement and sensitivity in bio-imaging applications, with the added potential for performing multi-modal imaging [4]. In 1995, Doxil^®^ (Sequus Pharmaceuticals Inc.) - a liposomal formulation of the chemotherapy drug doxorubicin, became the first Food and Drug Administration (FDA) approved cancer nanomedicine. Since then, nine cancer nanomedicines [5] and two nano-imaging agents [6] have received approval. The most recent is Vyxeos™ (Jazz Pharmaceuticals plc.), the first approved dual-drug liposomal nanoparticles, which are loaded with daunorubicin and cytarabine (at a proven synergistic 5:1 ratio). Vyxeos delivers drugs to bone marrow to treat acute myeloid leukaemia (AML), and was shown to successfully improve the overall survival rate of newly diagnosed AML patients. This result is encouraging and continues to drive the design, synthesis, and application of NDDSs for cancer nanomedicine [7].

Despite the myriad of NDDSs that have been synthesized and exploited by researchers over the last decade, only about 2% have progressed into clinical trials [8]. Thus, an obviously large discrepancy exists between nanomedicine research and its clinical translation [8]. Factors preventing nanomedicines reaching the clinic include a poor understanding of the dynamic and often limiting interactions that occur between NDDSs and the human body, referred to as the “bio–nano” interactions [2]. This typically reduces the accumulation of NDDS at their site-of-action (e.g., tumors) which significantly diminishes their therapeutic efficacy [9]. Chan et al. recently conducted a multivariate analysis of NDDSs’ preclinical studies over a 10-year period, finding that only 0.7% (median value) of the injected dose of an NDDS was detected in tumor tissue in vivo [9]. Additionally, scaling-up the synthesis/production of NDDS for use in large clinical trials also presents an ongoing challenge to the nanomedicine field [3]. Therefore, a successful implementation of NDDSs in cancer nanomedicine requires new and easily manufactured materials that can work on “bio–nano” interactions inside patients.

Protein-based nanoparticles (PNPs) PNPs are composed of protein subunits that possess the remarkable ability to spontaneously and precisely self-assemble into nanoparticles with hollow interior cavities. In nature, these internal cavities act as reaction chambers for enzymes; storage containers for inorganic materials; scaffolds for partially unfolded proteins; and protective nanocarriers for genetic material (e.g., viral DNA). Accordingly, PNPs play critical roles in all domains of life and can generally be classified as virus particles (VPs), virus-like particles (VLPs), non-viral particles (NVPs), or a de novo PNPs [10,11]. This review focus solely upon VLPs that are derived from viruses and NVPs that are derived from prokaryotic (e.g., bacteria) or eukaryotic (e.g., plants) sources (as listed in Table 1).

PNPs have a number of intrinsic properties that make them attractive alternatives to the synthetic nanoparticles used in conventional NDDSs [12,13]. PNPs are amenable to both genetic and chemical modification and have three distinct components with functionalities that lend themselves to applications in drug delivery: (i) an interior cavity that can be loaded with therapeutic cargo (e.g., small molecule drugs); (ii) an exterior surface that can be functionalized to display cancer-targeting ligands for drug targeting; and (iii) interfaces between the their protein subunits which can potentially be engineered to allow disassembly for controlled drug release. Additionally, PNPs are non-toxic and biodegradable, which is essential in biomedical applications [10,11]. Since PNPs are synthesized within biological systems, they require fewer production steps and show low inter-batch variability during the manufacturing process [14].

In this review, we describe PNPs’ “bio–nano” interactions and the existing strategies employed to engineer them into effective NDDS for cancer nanomedicine. We also discuss future directions for PNP research and what challenges must be addressed to successfully translate them into the clinic.

## 2. Current Clinical Status of PNPs

Of all the reported PNPs, VPs and VLPs have had the most success in the clinic. This is due to their natural capacity to infect and deliver nucleic acids, which has enabled them to be readily repurposed as vehicles for gene therapy, oncolytic viral therapy, and cancer immunization. In 2003, Gendicine^®^ (Sibiono GeneTech Ltd., Shenzhen, China) became the first FDA-approved VP for gene therapy treatment of head and neck carcinoma. Gendicine is a human adenovirus carrying the p53 tumor suppressor gene which induces cell-cycle arrest, senescence, apoptosis, and/or autophagy in cancer cells [40]. In 2015, the FDA approved Talimogene laherparepvec (T-VEC or Imlygic™) as the first oncolytic virus therapy, which is used to treat patients with advanced melanoma [41]. T-VEC is a genetically engineered variant of an oncolytic herpes virus (155–240 nm) that contains double strand DNA in which some viral genes have been deleted and the gene encoding human granulocyte-macrophage colony-stimulating factor (GM-CSF) was inserted [42]. T-VEC is applied locally to induce viral replication inside tumor cells, causing cell destruction and stimulating an immune response against melanoma cells [41]. Other successful PNPs include vaccines that prevent the sexually transmitted oncovirus human papillomavirus (HPV), which is the primary cause of cervical cancer. For example, the Gardasil 9^®^ (Merck) vaccine is a VLP composed of various capsid protein sub-units from different HPV types. This recombinant VLP lacks any viral DNA cargo and is, therefore, non-infective and cannot induce cancer. It does, however, trigger the generation of HPV-specific antibodies that protect vaccinated patients from becoming infected and acquiring HPV-related cancers [43]. Despite these successes, the overall use and clinical translation of PNPs is limited, with most PNP-related research languishing at pre-clinical stages.

## 3. Engineering PNPs as NDDS

**Figure 1:**
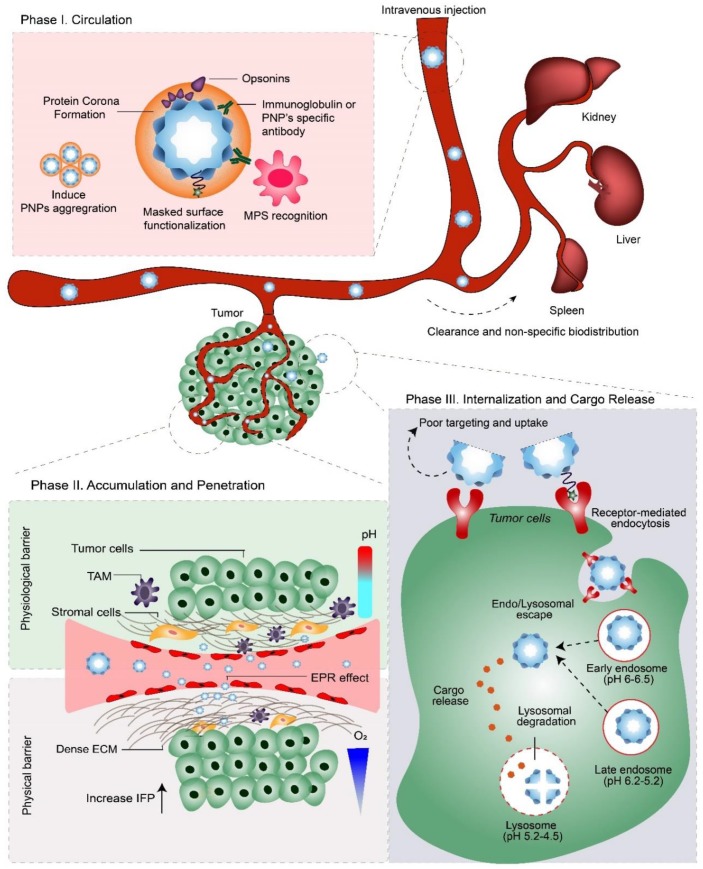
The three phases of PNP delivery and the pathophysiological barriers they encounter after systemic administration (i.e., intravenous injection). Phase I: ‘Circulation.’ After entering the bloodstream, protein corona formation on PNPs can evoke recognition by the mononuclear phagocytic system (MPS), leading to their clearance via MPS-rich organs; e.g., liver, kidney, and spleen. Therefore, to enable therapeutic concentrations of the NDDS to interact with tumors, PNPs must evade the MPS, while retaining their stability during blood circulation. Phase II: ‘Accumulation and Penetration.’ PNPs must extravasate from the bloodstream via leaky vasculature into the tumor, where they must overcome physical and physiological barriers to penetrate deep into the tumor microenvironment. Phase III: ‘Internalization and Cargo Release.’ Following endocytosis, PNPs are trafficked into endo/lysosomes and finally degraded to release their therapeutic cargo. Depending on their cargo’s mode-of-action, PNPs may need to escape (and avoid degradation) from endo/lysosomal compartments to the cytosol. TAM= tumor-associated macrophages; EPR effect = enhanced permeability and retention effect; ECM = extracellular matrix; IFP = interstitial fluid pressure.

**Figure 2:**
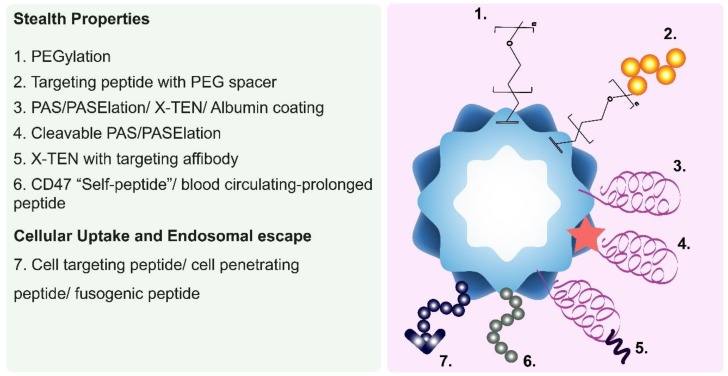
Illustration showing the various outer surface modifications used to make ‘stealth’ PNPs, and/or enhance their cellular targeting, uptake, and intracellular trafficking.

Ideally, a systemically administered NDDS must exhibit long blood circulation times, evade recognition and clearance by the immune system, extravasate from blood-vessels via enlarged endothelial gaps into tumors, penetrate through dense stroma and accumulate within the complex tumor microenvironment, selectively enter tumor cells, and finally release an active agent to induce a therapeutic effect. This journey and the eventual fate of an NDDS inside the human body was recently defined by Sun et al. as the “CAPIR Cascade”—circulation, accumulation, penetration, internalization, and release [44]. In this section, we highlight the in vivo behavior of systemically delivered PNPs (Figure 1) and discuss the potential solutions used to overcome the challenges PNPs face during each phase of the CAPIR Cascade (Figure 2 and Table 2).

### 3.1. Phase I: Circulation

Systemically administered PNPs often display short blood circulation times and rapid clearance in animal models, which has significantly impeded their advancement as NDDSs [45]. For example, simian virus 40 (SV40) was eliminated from circulation only 5 min after IV injection in mice [25], while cowpea mosaic virus (CPMV) was cleared after 30 min, with a half-life of 4–7 min [46]. Studies with the tobacco mosaic virus (TMV) showed a circulation half-life of 30 min [47], whereas potato virus X (PVX) was cleared after 2–6 h with a half-life of 12.5 min [48]. Moreover, many of these PNPs were found to accumulate in organs of the mononuclear phagocytic system (MPS), such as the liver, spleen, and kidneys [48]. The half-life of cowpea chlorotic mottle virus (CCMV) and heat shock protein (Hsp) have not been reported, but studies have shown that both were broadly distributed throughout the body and rapidly excreted from the mice [49]. These poor pharmacokinetic and biodistribution profiles are driven by different factors, but primarily the formation of protein coronas on PNP surfaces.

In the first phase of systemic delivery, PNPs enter the blood stream, wherein plasma proteins adsorb non-specifically onto their surfaces to form a biologically active coating termed the “protein corona” [50]. The formation of this corona is unavoidable, rapid and dynamic, and is primarily determined by the PNPs’ surface chemistry, charge, and size [50,51]. The biological fate and function of a PNP is the result of specific physiological responses toward this ‘PNP–protein corona’ complex (Figure 1, Phase I). Importantly, opsonin proteins (e.g., immunoglobulins (IgG) and complement proteins) within the protein corona trigger recognition by the MPS, which promptly engulfs any opsonin-bearing PNPs and clears them from the bloodstream. This fast-acting innate immune response reduces PNP concentrations, preventing therapeutic amounts reaching the site of action and thus represents a significant barrier to success for many NDDS.

Protein corona formation on PNPs remains understudied. In the only detailed report on this topic, Steinmetz and co-workers showed that a protein corona formed on the surface of TMV after exposure to blood plasma [16]. The amount of proteins adsorbed onto TMV was about six-fold lower than that those absorbed onto synthetic silica oxide nanoparticles. This less efficient adsorption of proteins was due to the TMV’s heterogeneous surface properties (i.e., variable charge and hydrophobicity), while the synthetic silica oxide nanoparticles’ smooth surfaces and uniform surface charges promoted the formation of a rich protein corona. Opsonins (i.e., IgG and complement protein C3) were the most abundant components of the protein corona of TMVs [16], making clearance by the MPS likely. Furthermore, heat-induced changes in TMV’s morphology from rod-shaped to spherical, and the incorporation of lysine residues to increase its surface charge positivity, had only minimal effects on TMV’s protein corona profiling [16]. Uptake of PNPs by macrophages may also induce the adaptive immune response that generate PNPs-specific antibodies, which also contribute to the rapid elimination of PNPs in the bloodstream. For example, Aanei et al. reported that the repetitive injection of MS2 protein nanocages into mice resulted in the generation of IgG, IgM, and MS2-specific antibodies. The MS2-specific antibodies were also detected despite the presence of protective polyethylene glycol (PEG) coatings on the MS2 surface [52]. Similar results were also observed for TMV, in which TMV-specific antibodies were generated after a second injection of both native and PEGylated-TMV in mice [17].

The formation of a protein corona can affect a PNP’s colloidal stability and biodistribution profile [16]. When PEGylated-TMV was decorated with a fibrinogen-binding peptide (GPRPP), the resulting TMV–PEG–GPRPP rapidly aggregated after exposure to human plasma, while TMV–PEG remained stable. GPRPP peptide has strong affinity towards fibrinogen; therefore, increasing the proportion of fibrinogen within the protein corona of TMV–PEG–GPRPP, which could be responsible for the observed aggregation [16]. Following injection into a mouse model, TMV–PEG–GPRPP displayed higher accumulation in the lung than TMV–PEG, due to aggregation and filtration through lung capillaries. Thus, as shown in this study, the degree of PNP aggregation may affect its bio-distribution profile in vivo.

#### Enhancing the Pharmacokinetic and Biodistribution Profiles of PNPs

To be considered a suitable NDDS for clinical applications, a PNP must demonstrate good in vivo pharmacokinetic and biodistribution profiles. Accordingly, a PNP needs be resistant to degradation and metabolism, evade recognition and clearance by the immune system, and avoid non-specific uptake by off-target cell types (e.g., endothelial cells). To address these challenges, a variety of “stealth” coatings have been developed and applied to PNPs, some of which we discuss below.

- PEGylation

PEGylation is the most well-established and practical strategy to help NDDSs evade the immune system and increase their blood circulation times. PEGylation reduces serum protein adsorption and opsonization, while promoting the formation of protein coronas enriched with dysopsonins. Dysopsonin proteins (e.g., clusterin/ApoJ) inhibit phagocytic ingestion by macrophages [53]. Recently, the PEGylation of TMV was shown to reduce opsonization, with less complement proteins, plasminogen and immunoglobulins observed within their protein coronas [16]. This altered corona probably explained the doubled circulation time of the TMVs from 3.5 min to 6.3 min [47]. In another study, PEGylated SV40 showed 100-fold lower internalization by RAW264.7 murine macrophage cells, which reduced its distribution within MPS organs (i.e., liver, spleen, and bone marrow) and prolonged its in vivo half-life from 5 min to 12 h [25].

Despite its widespread use, PEG coatings have been shown to trigger the generation of PEG-specific antibodies after repeated administration, leading to the clearance of PEGylated entities from blood (also referred to as the ‘accelerated blood clearance (ABC) phenomenon’) [53]. This new observation has led to the search for alternative ‘stealth’ coatings for NDDSs, including PNPs. Besides PEG, several anti-fouling polymers have been reported as potential stealth coatings for NDDS, like polybornene, polypyrrole, poloxamer, and many others [54,55,56].

- Serum Albumin Coatings

Albumin is a naturally abundant plasma protein that can be used as a surface coating to avoid the PEG-specific antibody clearance of PEGylated-PNPs. For example, serum albumin (SA) coatings were covalently cross-linked to PEGylated-TMV (SA/PEG–TMV) [57]. When compared to PEGylated-TMV, in vitro dot blot studies revealed that SA coatings made SA/PEG–TMV 5–6 times less recognizable by both TMV and PEG-specific antibodies. However, despite reduced recognition by antibodies, SA/PEG–TMV was internalized by RAW294.7 macrophages in vitro in a similar manner as PEGylated-TMV. Moreover, after repetitive injection, balb/c mice treated with PEGylated-TMV or SA/PEG–TMV still showed a robust production of TMV-specific antibodies, but no detectable amounts of SA-specific antibodies. In vivo studies also showed that the SA/PEG–TMV had a 100-fold greater half-life than PEGylated TMV. This study indicated that additional SA coating can mask the TMV from recognition by both TMV and PEG-specific antibodies. This was confirmed by the negative binding of the TMV-antibody isolated from SA/PEG–TMV immunized mice towards SA/PEG–TMV in dots blot study [17,58].

In a similar study, ferritin was genetically modified to display a serum albumin-binding domain (from the streptococcal protein G) on its external surface (ABD–Hfn), enabling the facile non-covalent attachment of serum albumin coatings upon entering the blood plasma [26]. ABD–Hfn was loaded with DOX as a drug model and fluorescent reporter in pharmacokinetic studies performed in mice. When compared to wild-type ferritin, ABD–Hfn successfully extended DOX’s blood circulation half-life (from 0.9 h to 17 h) and residence time (from 1.97 h to 17 h) [26].

- Blood circulation-prolonging peptides

A biomimicry strategy to avoid clearance by the immune system is to attach the CD47 “do not eat me” signal to NDDS. The cell-surface protein CD47 interacts with its receptor on macrophages, SIRPα, to prevent the phagocytosis of normal ‘self’ cells. The critical segment of the CD47 protein, referred to as the ‘self-peptide,’ has been used as a functional anti-phagocytosis ligand for NDDS [59]. For example, presentation of the ‘self-peptide’ on the surface of liposomal [60] and polystyrene nanobeads [59] reduced their macrophage-mediated clearance, thus enhancing their circulation times and accumulation in tumors. This approach has also been applied to PNPs, for instance, the P22 nanocage was genetically engineered to display the ‘self-peptide’ on its outer surfaces (via its ‘decoration protein’). This modification reduced P22 nanocage uptake by primary splenocytes in vitro by 50% [35]. However, biodistribution and pharmacokinetic studies are needed to confirm the enhancing effect the ‘self-peptide’ has on the circulation half-life of P22 nanocages in vivo [35].

Another reported peptide that extends the half-life of PNPs is the blood circulating-prolonged peptide 1 (BCP1) which is derived from the MS13 bacteriophage [61]. BCP1 was genetically fused at the N-terminus of human heavy chain ferritin which was then loaded with DOX. In mice, the DOX-loaded BCP1-ferritin had a half-life of 14.9 h, whereas the half-lives of DOX-loaded wild-type ferritin and free DOX were only 7.8 h and 3.3 h, respectively. BCP1 was also shown to delay macrophage-mediated clearance and enhance blood retention ferritin due to its binding interactions with platelets. Due to its extended circulation time, DOX-loaded BCP1-ferritin showed greater antitumor activity in vivo than DOX-loaded ferritin or free DOX alone [61]. Interestingly, the interactions between BCP1 and ferritin could be potentially exploited in ‘cell-mediated delivery’ strategies, wherein PNPs are modified to bind the surfaces of living cells (e.g., platelets, red blood cells or leukocytes) to increase their circulation time and/or targeting efficiencies [62,63].

- Long Repetitive Hydrophilic Peptides

Repetitive and highly hydrophilic peptides have been designed to mimic the physicochemical and ‘stealth’ properties of PEG. Falco and co-workers used ‘PASylation,’ a technique in which they genetically fused PAS (Pro–Ala–Ser) polypeptides at the N-terminus of ferritin subunits (up to 75 residues in length). PASylation was used to prevent the interaction of ferritin and its natural receptor TfR-1, reducing its uptake by healthy off-target cells and prolonging its circulation time. Using surface plasmon resonance, the PASylated ferritin displayed an about six-fold lower binding affinity towards TfR-1 than wild-type ferritin [29]. In vivo, PASylated ferritin prolonged the DOX half-life up to 12.3 h, while the wild-type ferritin only up to 2.7 h [64]. The same research group also reported a modification of this peptide by adding glutamate (Glu (E)) residues to form a repetitive ‘PASE’ sequence. The Addition of glutamate was used to introduce a negatively charged residue into the modified ferritin, thus promoting a longer circulation lifetime and reducing accumulation in the liver [28]. While loaded with DOX, PASE-ferritin had a significantly higher plasma residence concentration, higher DOX accumulation in tumor tissue, and lower accumulation in the liver, compared to PAS-ferritin.

Kim and co-workers [32] developed long circulating ferritin nanocages (LCFNs) with “intrinsically disordered proteins” (referred to as X-TEN) as a stealth layer. X-TEN is a randomized amino acid sequence composed of hydrophilic amino acids (Ala, Gly, Glu, Pro, Ser and Thr). The genetic fusion of X-TENs of varying lengths to the outside of ferritin, resulted in a series of LCFN variants with enhanced in vivo pharmacokinetic profiles. For example, when compared to wild-type ferritin nanocages (wtFN), the LCFN variant LCFN144, displayed a ~12-fold longer circulation half-life (t_1/2_), ~13.5-fold greater mean blood residence time; and a 37-fold slower clearance rate (ml hr^−1^ kg^−1^) [32]. X-TEN coatings were also shown to reduce the internalization of LCFN144 by macrophages and endothelial cells, indicating their ability to mask LCFN144 from the MPS and prevent non-specific cellular uptake (by cells expressing natural ferritin receptors). In a tumor-bearing mouse model, these ‘stealth’ properties allowed LCFN144 to passively accumulate over time within tumors in vivo, which was further improved by the attachment of tumor-targeting affibodies, leading to a two-fold increase in tumor accumulation in vivo when compared to wtFN.

### 3.2. Phase IIa: Accumulation

In the next phase of delivery, PNPs must extravasate out of blood vessels into the tumor site (Figure 1, Phase II). By means of prolonged circulation times, PNPs can accumulate inside tumors via the enhanced permeability and retention (EPR) effect. The EPR effect relies on the differences between the vascularization of normal tissues and tumors. In normal tissues, small molecules transit easily out of the blood stream, while large molecules, such as NDDS, cannot. In contrast, the tumor vasculature is a chaotic network of undeveloped and “leaky” angiogenic blood vessels, which allows the extravasation of large NDDS that are up to several hundred nanometers in size. This, in conjunction with impaired lymphatic drainage, results in the relatively effective and somewhat selective accumulation of NDDS within tumors [9,65]. Accordingly, most FDA-approved cancer nanomedicines exploit the EPR effect to “passively” target and accumulate within diseased tissues. However, the clinical efficacy of the EPR effect has become highly controversial. This is due to widespread variations in the presence of leaky tumor vasculature and its inconsistent molecular cut-off size, which is dependent on the patient and the tumor’s location, stage, and type [66]. In addition, tumor tissue exhibits irregular blood vessel formation and permeability, poor blood flow, and non-uniform interstitial fluid pressure (IFP), which further limits the extravasation and perfusion of NDDSs [66,67]. Therefore, tactics to overcome these barriers are needed.

#### Improving the PNP’s Tumor Accumulation

A number of strategies have been developed to enhance the transit of NDDSs across blood vessels into tumors. These mostly involve modifying the physicochemical and functional properties of NDDSs, and/or modulating the tumor vasculature itself. In general, NDDSs that have small dimensions (12–60 nm) and are neutrally charged, elongated, and flexible, demonstrate good blood-vessel crossing [68]. Steinmetz and colleagues investigated the relationship between the aspect ratio and the passive targeting properties of PNPs [69]. Filamentous TMVs of varying lengths (Ls) and aspect ratios (ARs) were produced and subsequently functionalized with PEG. The resulting PEGylated TMVs were referred to as PEG-TL (L 300 nm; AR 16.5), PEG-TM (L 130 nm; AR 7) and PEG-TS (L 60 nm; AR 3.5) [69]. After injection into tumor-bearing mice, AlexaFluor647 labelled-PEG-TS showed the highest passive accumulation within tumor tissue (ex vivo fluorescence quantification). This result was due to PEG-TS’s small size and low AR, which enabled it to extravasate through the “leaky” tumor vasculature more effectively than the larger PEGylated TMVs with higher ARs [69]. This highlights the need to select of PNPs with favorable physical (size and shape) and functional (e.g., PEGylation) properties that enable extravasation.

Techniques that augment the physiology of the tumor vasculature have been used to enhance the EPR effect. These include, increasing blood flow by administration of vasodilator agents (e.g., nitric oxide); enhancing blood vessel permeability by physically disrupting tumor vasculature; and weakening a tumor’s structural strength and internal pressure by eliminating cells located within the tumor itself [70]. PNPs have also been designed to facilitate EPR enhancement. For example, Zhen et al. engineered photosensitizer-loaded RGD (Arg–Gly–Asp) modified ferritin (ZnF16Pc-loaded RFRT) that could “actively” target tumor vasculature (via the integrin-binding activity of RGD) [71]. After IV administration of ZnF16Pc-loaded RFRTs into mice bearing subcutaneous tumors, photodynamic therapy (PDT) was performed. In this process, light-activated ZnF16Pc-loaded RFRTs delivered destructive reactive oxygen species (ROS) to the tumor endothelium, which permeabilized tumor blood vessels and increased the ‘leakiness’ of the tumor vasculature. Systemically administered Doxil (DOX-loaded liposomes) were then shown to easily pass through the disrupted endothelial barrier and enter the tumor parenchyma, indicating EPR enhancement. With this pre-treatment step, 20-fold more Doxil accumulated in the tumor site and its therapeutic efficacy increased by 75% in vivo [71]. This study highlights the potential for engineered PNPs to directly modulate physiology and their compatibility with other NDDSs to create multimodal nanomedicine-based treatment regimens.

### 3.3. Phase IIb: Penetration

After extravasation from blood vessels, PNPs enter the highly complex and heterogeneous tumor microenvironment (TME) (Figure 1, Phase II). The TME is composed of cellular and non-cellular components. The TME possesses unique physiological conditions that are associated with tumor growth and progression, such as acidic pH; hypoxia; high interstitial fluid pressure (IFP); and proteinases (e.g., metalloproteinases), which are commonly used as a trigger for stimuli responsive NDDSs [72]. Malignant cells within solid tumors make up the tumor parenchyma, which are supported by the tumor stroma. The tumor’s stroma consists of a diverse range of elements, including new blood vessels, abundant tumor-associated macrophages (TAMs), connective tissue cells, and the extracellular matrix (ECM) [73]. Connective tissue cells like fibroblasts and mesenchymal stromal cells are highly active within the tumor parenchyma and produce a range of growth factors, cytokines, chemokines, and proteinases. They are referred to as “cancer-associated fibroblasts” (CAFs) and their activation directly promotes the proliferation of tumor cells, tumor angiogenesis, and ECM remodeling. The ECM is a structural scaffold built by fibrous molecules, such as collagen, hyaluronic acid and proteoglycans. During its remodeling, the ECM becomes denser, more rigid and more fibrotic. This increases stress on the solid tumor and compresses its vasculature, thus becoming a significant physical barrier and limiting factor for the movement, penetration, and distribution of an NDDS inside the tumor parenchyma [73].

The smaller the size of the NDDS, the greater its ability to transport and diffuse through tumor interstitial space. Based on the same principles for enhancing extravasation, tumor penetration by PNPs is greatly influenced by their size, shape, and surface charge. Therefore, due to the inherently small dimensions (as low as ~10 nm) of most PNPs they are able to move through the extracellular space within the TME via simple diffusion. Similar to synthetic NDDSs, filament-like PNPs show better abilities to penetrate deeper and diffuse through fibrous matrices (like collagen) due to their physical flexibility [74]. For example, fluorescent microscopy was used to visually track fluorescently-labelled filamentous PVX in an avian embryo tumor xenograph model. PVXs were observed accumulating, penetrating, and finally reaching the tumor’s inner core. In contrast, fluorescently-labelled spherical CCMVs were seen spreading throughout the tumor without any enhanced localization in the inner core region. Similarly, in tumor-bearing mice, PEGylated PVXs showed enhanced tumor tissue penetration and 15% higher tumor accumulation than PEGylated CPMV [74]. Thus, the physical properties of PNPs should be considered when delivering therapeutics to solid tumors.

**Figure 3:**
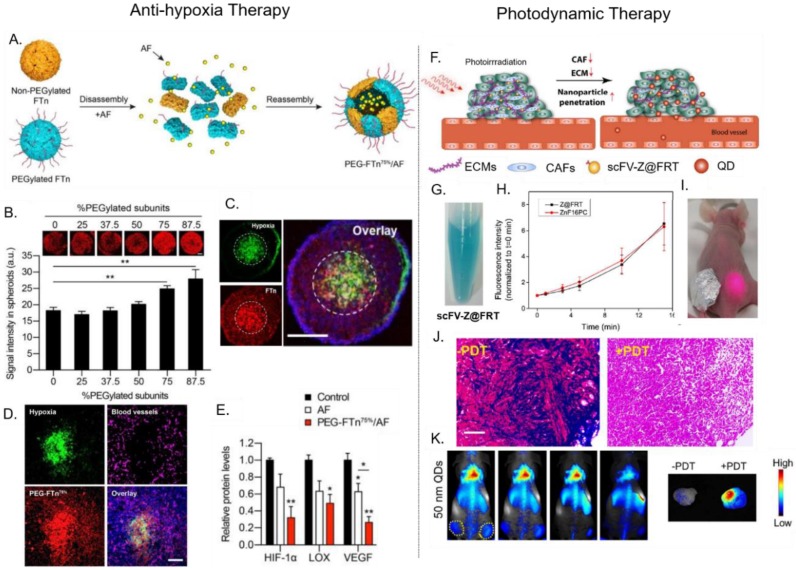
Examples of PNP-mediated drug delivery for different cancer therapies. (**Left panel**) Anti-hypoxia therapy: ferritin-mediated delivery of the HIF-1α inhibitor, Acriflavine (AF). (**A**) AF is encapsulated within PEGylated ferritin (FTn) via assembly-disassembly method. (**B**) Fluorescence microscopy showed that the PEGylation density of FTn affected its ability to penetrate 3D lung cancer tumor spheroids. (**C**) FTn with a PEG coverage of ≥75% (PEG–FTn^75%^) showed deep penetration and accumulation within inner hypoxic areas of tumor spheroids. (**D**) Tumor sections from orthotropic lung tumor-bearing mice showed that PEG–FTn^75%^ successfully penetrated inner hypoxic areas in tumors after systemic injection. (**E**) Delivery of AF using PEG–FTn^75%^ significantly reduce HIF^5^-1α, LOX, and VEGF expression in vivo. Adapted from [27], with permission from American Chemical Society, 2019. (**Right Panel**) Photodynamic therapy (PDT): ferritin-mediated delivery of photosensitizers for photodynamic therapy. (**F**) Ferritin is used to target the delivery of photosensitizers into tumors to mediate PDT which kills cancer-associated fibroblasts (CAFs), weakens the extracellular matrix (ECM), and enhances the penetration of nanoparticles into the tumor. (**G**) ZnF16PC-loaded chain variable fragment (scFv)–ferritin for targeted PDT (**H**) Both the free photosensitizer ZnF16PC and ZnF16PC-loaded scFv–ferritin produced similar amounts of reactive oxygen species ROS upon irradiation in time dependent manner (**I**) Bilateral tumor bearing mice were used to compare the effect of PDT on the reduction of collagen level in the tumor ECM. The tumor covered with aluminum foil was not irradiated. (**J**) Tumor section from irradiated tumor and non-irradiated tumor. ECM levels were decreased by PDT in the irradiated tumor (Mason’s trichome staining). (**K**) Increased accumulation of 50 nm quantum dots (QDs) in the irradiated tumor after PDT indicated that the decrease in ECM improves the penetration and accumulation of the NDDS in tumor tissue (Maestro II imaging system). Adapted from [31], with permission from American Chemical Society, 2018.

One strategy to enhance the tumor penetration of some PNPs is to exploit their natural affinity toward cell-surface receptors, which can facilitate receptor-mediated transcytosis/transcellular transport deep inside tumors. This phenomenon was recently shown between ferritin (FTn) and its native Transferrin receptor-1 (TfR-1). Huang et al. [27,75] performed tumor penetration studies using 3D lung cancer cell spheroids. Bare FTn and FTn PEGylated with low molecular weight PEG (FTn–PEG2K) distributed uniformly throughout the entire spheroid (including its inner core), while FTn PEGylated with higher molecular weight PEG (FTn–PEG5K and FTn–PEG10K) were predominately localized in the spheroid’s periphery. Interestingly, in the presence of anti-TfR-1 (blocking agent), FTn–PEG2k became unable to penetrate the tumor, localizing in the outer regions. These results indicated that TfR-1 played an important role in mediating FTn tumor penetration, while higher molecular weight PEG (long chain length) can hinder FTn/TfR-1 interactions, limiting FTn penetration. TfR-1 was overexpressed in the hypoxic and hard-to-reach interior regions of tumors. The authors also investigated the effect PEGylation coverage (%) had on the ability of FTn to penetrate lung tumor spheroids. In comparison to various PEGylated FTns, the FTn containing ≥75% PEGylated subunits (PEG–FTn^75%^ and PEG–FTn^85%^) displayed the greatest accumulation within the hypoxic interior of the spheroid model (Figure 3B). Following intratumoral injection into a tumor-bearing mice model, PEG–FTn^75%^ accumulated more efficiently within hypoxic regions of the tumor than non-PEGylated FTn. These studies indicate that the molecular weight and density of a PNP’s PEG coating (and other ‘stealth’ coatings) must be finely tuned to enhance a PNP’s circulation half-life while maintaining their ability to target and penetrate tumors [27,75].

### 3.4. Phase III: Internalization and Cargo Release

After the PNPs’ penetration and subsequent accumulation within the TME, their therapeutic cargo can be released either extracellularly within the TME or intracellularly inside the tumor cells (Figure 1, Phase III). This release can be triggered by environmental stimuli (extra or intracellularly) or by simple degradation of the PNP itself [12]. The desired release method is obviously contingent upon the cargo’s therapeutic mode-of-action.

For intracellular delivery, the outer surfaces of a PNP can be decorated with cancer-targeting ligands (e.g., peptides, aptamers, and antibodies) that mediate its selective recognition and uptake by tumor cells. However, ligands on a PNP surface are often sterically masked by a protein corona that forms during circulation. This causes non-selective binding and uptake of the PNP by otherwise healthy cells and tissues. In addition, the presence of stealth coatings that reduce corona formation, like PEG, may hinder interactions between the targeting ligand and its corresponding receptor, further reducing PNP uptake. This phenomenon is termed the “PEG dilemma.”

To overcome the PEG dilemma, active-targeting moieties are often conjugated to PNP surfaces via flexible spacer regions. For example, Steinmetz and co-workers incorporated PEG spacers between the surface of TMV and tumor-targeting RGD peptides [16]. Herein, PEG spacers of varying chain lengths (4, 12, or 24 PEG units) were attached to TMV, providing 25% PEG–RGD coverage in a ‘mushroom conformation.’ The longer the PEG spacer’s chain length, the more effective it was at mitigating TMV’s opsonization (‘stealth’). RGD-modified TMVs with PEG spacers displayed three times higher uptake in HeLa cervical cancer cells than those without PEG spacers. Thus, the PEG spacers improved the orientation of RGD, promoting its specific binding interactions with integrins overexpressed on the HeLa cells.

Cleavable-stealth coatings are another approach employed to address the PEG dilemma. For instance, ferritin was decorated, via genetic engineering, with a matrix metalloproteinase (MMP)-cleavable PASE peptide and loaded with the anticancer drugs DOX or mitoxantrone (via a disassembly–reassembly procedure) [28]. The PASE peptide acted as a stealth coating that prolonged ferritin’s half-life by reducing its unspecific uptake by normal cells. Once ferritin entered the TME, the PASE peptide was cleaved by intratumoral MMP-2. As a result, the unmasked ferritin was internalized via its natural transferrin receptor-1 (TfR-1 or CD71), which is overexpressed on cancer cells. Other physicochemical properties of the unique TME (e.g., acidic pH, hypoxia, and proteinases) can be exploited to trigger the cleavage of stealth coatings.

Like most NDDS, PNPs are internalized by tumor cells through a process known as endocytosis. Endocytosis pathways can be subdivided into four categories; namely, macropinocytosis, the clathrin-mediated pathway, the caveolae-mediated pathway, and the clathrin/caveolae independent pathway. The internalization of PNPs by tumor cells can occur via one or more of these pathways. Which pathway is activated is determined by a PNP’s size, shape, surface chemistry, and its interactions with the cellular membrane [76]. Most actively targeted PNPs enter cells via the clathrin-mediated pathway which is a receptor-mediated pathway. Except for the caveolae-mediated pathway, that may delivery the endosomal cargo to the cytosol, all three other pathways will first traffic a PNP to early endosomes (pH 6.5–6.0); then to late endosomes (pH 6.2–5.2); and finally, to lysosomes (pH 5.2–4.5). The harsh acidic pH and proteases (e.g., cathepsin) within lysosomes are then responsible for the degradation of PNPs and/or their cargo [77].

PNPs have been reported to demonstrate different intracellular fates upon endocytosis. Wild type SV40 entered CV-1 cells (kidney murine cells) via the caveolae-mediated pathway and were then transported to the cell’s endoplasmic reticulum (ER) [78]. CPMVs were shown to be taken up by HeLa cells through both the caveolae-mediated pathway and micropinocytosis, resulting in their accumulation inside late endosomes and lysosomes [79]. Chang et.al reported that the MS2 bacteriophage decorated with an epidermal growth factor receptor (EGRF)-targeting peptide, GE11, was internalized by Hep2G (hepatocellular carcinoma) cells via both the clathrin-mediated and micropinocytosis pathways [80]. Moreover, the GE11-decorated MS2 was internalized by the cells within 30 min and reached maximum intracellular levels after 12 h [80]. However, this study did not specify whether GE11-MS2 ended up inside lysosomes. In the majority of studies, PNPs are localized inside lysosomes following their endocytosis, leading to their degradation. This degradation can be potentially utilized as a mechanism to release small-molecule chemotherapeutic drugs; e.g., DOX.

In most cases, PNP degradation is not desirable; PNPs should, therefore, be designed to escape from endo/lysosomes. A proven strategy to promote PNPs’ endosomal escape is to modify their surfaces with cell-penetrating peptides (CPPs) and/or fusogenic peptides. CPPs are highly cationic and amphiphilic peptides that can interact with membrane lipids [81]. The TAT peptide (GRKKRRQRRRPQ)—derived from the transactivator of transcription (TAT) of the human immunodeficiency virus, is a widely used CPP, and has been previously attached to MS2 and TMV to enhance endosomal escape in siRNA delivery applications [15,20]. M-lycotoxin peptide L17E which is derived from spider venom, is another CPP capable of disrupting endo/lysosomal membranes [23]. L17E was conjugated to CCMV and used to enhance the cytosolic delivery of GFP siRNA in an in vitro model. The resulting L17E–CPMV successfully increased siRNA transfection efficiency and was able to ‘silence’ GFP expression by 50% in genetically engineered HeLa cells. In contrast, unconjugated CPMV decreased GFP expression by 30% in the same model, indicating L17E’s ability to mediate the endosomal escape of PNPs.

In another report, Ashley et al. genetically modified MS2 to display the SP94 peptide—which selectively targets hepatocellular carcinoma (HCC) cells. The resulting MS2–SP94 was internalized by HCC cells, but became trapped inside lysosomal compartments where it can be degraded. To avoid this, MS2–SP94 was additionally engineered to co-display the H5YGW fusogenic peptide. The histidine-rich H5YGW peptide became protonated by the endosome’s acidic pH (pKa = 6.0), which induced the osmotic swelling of endosomes and the destabilization of endosomal membranes [19]. MS2 co-displaying SP94 and H5YGW were observed to be taken up by HCC cells, and subsequently distributed inside the cytosol 1 h after their endocytosis, thus indicating their ability to escape from endosome [19]. Importantly, this work shows that the intracellular fate of PNPs can be tuned to best deliver its therapeutic cargo.

## 4. The Application of PNPs as NDDS for Cancer Therapeutics and Imaging Agents

PNPs are used as nanocarriers for anticancer drugs and imaging agents. A variety of methods to load drugs inside PNPs have been reviewed elsewhere [12]. Different passive and active targeting methods have been tested to help the delivery of drug-loaded PNPs to cancers. In this section, we describe current methods available for designing PNPs as NDDS for cancer therapy and imaging.

### 4.1. The Delivery of Anti-Cancer Therapeutics

Encapsulation of a drug by a PNP should improve a drug’s physiochemical and pharmacokinetics properties. A good design would also increase drug loading, maintain the stability of both the PNP and its cargo, and minimize early drug leakage during storage and en route to its site-of-action. Furthermore, PNPs made for clinical purposes should be reliable, scalable, and compatible with other clinically compliant processes.

Ferritin is the most commonly used PNP for the delivery of chemotherapeutics in research. Ferritin disassembles in acidic pHs or high salt conditions, and is then made to reassemble in the presence of a drug at a neutral pH or in normal salt conditions, respectively. The drug becomes encapsulated within its interior cavity. Although ferritin as a drug loading method has limitations, this phenomenon has been exploited by researchers to encapsulate chemotherapy agents; encapsulation of doxorubicin (DOX) in each ferritin particle via the pH-mediated disassembly/reassembly method resulted in the uptake of 20 to 30 DOX molecules. However, 30% of the drug was lost via leakage during the first week of storage [64]. In addition, 50% of ferritin PNPs were lost because of acid-induced precipitation. The reassembly of ferritin is not fully reversible because losses in protein subunit conformation can cause DOX leakage [83]. We should mention that losing DOX because of hydrolysis after exposure to low pH was not investigated by the authors.

Hypoxia-inducible factor 1-alpha (HIF-1α) is a transcription factor that mediates adaptive responses to hypoxia and plays important roles in tumor growth and progression [84]. Due to ferritin’s inherent ability to reach hypoxic areas within solid tumors (where HIF-1α is upregulated), PEGylated ferritin (PEG–FTn^75%^) has been utilized to target the delivery of the HIF-1α inhibitor, acriflavine (AF) (Figure 3A) [27]. AF was first encapsulated inside PEG–FTn^75%^ using a denaturant triggered disassembly-reassembly method, resulting in 62 ± 4 AF molecules per PEG–FTn^75%^. Compared to free AF, AF-loaded PEG–FTn^75%^ showed a four-fold longer circulation time (mean residence time: 1.39 h versus 5.91 h) and a 72-fold greater area under curve (AUC). This enhanced pharmacokinetic profile resulted in seven-fold higher AF accumulation in tumor tissue in vivo, after intravenous administration. Fluorescent imaging of 3D lung cancer cell spheroids and in vivo tumor sections showed that PEG–FTn^75%^ successfully penetrated into inner hypoxic areas in these tumor models (Figure 3B,C). Consistent with this result, PEG–FTn^75%^ that was systemically delivered into an orthotropic lung cancer mouse model, was shown to accumulate in the hypoxic areas of tumors that lacked vascularization (Figure 3D). Further, using real time PCR and western blot analysis, PEG–FTn^75%^/AF was also found to be two times more effective at inhibiting the in vivo expression of HIF-1α and HIF-1α driver genes (i.e., vascular endothelial growth factor, VEGF), when compared to free AF (Figure 3E). This study comprehensively demonstrates that a drug can be efficiently loaded into PEG–FTn^75%^, resulting in improved pharmacokinetic profiles, targeted delivery (i.e., inner hypoxic regions), and enhanced efficacy.

As an alternative to the well-established pH- or denaturant-mediated disassembly/reassembly method, Zhen et al. [85] exploited the native divalent metal (i.e., Fe(II)) binding functionality of ferritin to load it with drugs. By pre-complexing metal copper ion with DOX (Cu-Dox), the DOX loading of RGD-modified ferritin reached ~30 wt%. Ahn et al. [30] further optimized a ‘nicked ferritin,’ a hybrid ferritin composed of human heavy chain ferritin and four-fold truncated ferritin (F-160). This nicked ferritin variant had wider pore openings than the original (<4Å) and displayed increased Fe(II)-Dox loading. This strategy successfully provided 70% protein stability after incubation of the ferritin with metal. Through this method a DOX final load of 121 molecules per PNPs was achieved which remained stable in neutral pH for over 25 h [30]. In another drug-loading method, Wang et al. [86] tested high hydrostatic pressure (500 MPa) at a pH of 5.5 to disassemble ferritin and to increase protein conformation recovery. Following disassembly at high pressure, a gradual decompression was used to induce gently ferritin reassembly and DOX encapsulation. Protein aggregation was reduced by the addition of 20 mM arginine during this process and resulted in 100% ferritin recovery and no premature drug leakage over 2 weeks. This approach may provide a method for the scaled-up production of drug encapsulation inside ferritin.

Other PNPs have been loaded with anticancer agents. Murata et al. [36] engineered the 13 nm small heat shock protein 16.5 (Hsp 16.5) PNP from the thermophilic archaeon *Methanococcus jannaschii* to deliver OSU03012 for the treatment of pancreatic cancer. OSU03012 is a novel derivative of celecoxib, which causes tumor cell death via endoreticulum stress-induced cell death signaling. Due to its hydrophobicity, OSU030312 could be physically entrapped within the hydrophobic regions of Hsp. To achieve this, OSU030312 and Hsp were mixed for 30 min at 50°C, which led to the encapsulation of 40–50 OSU030312 molecules per Hsp. To enable its targeted delivery, Hsp was also genetically modified to display iRGD—a peptide that binds the neurophilin-1 receptor overexpressed on pancreatic cells. For in vitro cell viability studies with AsPC-1 pancreatic cancer cells, OSU030312-loaded iRGD-Hsp (IC_50_ = 4.7 µM) was found to be slightly more cytotoxic than OSU030312-Hsp (IC_50_ = 5.4 µM) and up to two-fold more cytotoxic than free OSU030312 (IC_50_ = 10 µM). These results show that the Hsp PNPs are able to enhance both the delivery and efficacy of OSU030312.

Targeting ligands, therapeutics and fluorescent dyes can be covalently attached to reactive amino acid residues that are presented either naturally or by mutation on the PNPs surface. For example, Moon et al. [34] recently used this approach to modify the encapsulin protein nanocage from the bacterium *Thermotoga maritima* for drug delivery. The authors used amine-reactive chemistry to conjugate the hepatocellular carcinoma-targeting peptide SP94 to the naturally-occurring lysine residues on the exterior surface of the encapsulin, thus enabling their specific tumor-targeting capabilities. Encapsulin sub-units were genetically engineered to display a single cysteine residue, resulting in a total 60 surface-available cysteines. This enabled the precise use of sulfhydryl-reactive chemistry to covalently attach the 60 molecules of the anticancer prodrug, Aldoxorubicin (ALDox). The Aldox-SP94 encapsulin was shown to release 60% of the attached Aldox at pH 5 after 8 h. Furthermore, in vitro cytotoxicity studies using Hep2G hepatocellular carcinoma cells indicated that the Aldox-SP94 encapsulin showed similar toxicity to free Aldox. Thus, encapsulins, like other PNPs, can be readily engineered for the precise incorporation of functionalities, which is highly advantageous in clinical applications.

### 4.2. Delivery of Photosenstizers for Photodynamic Therapy (PDT)

Photodynamic therapy (PDT) can provide minimal invasive cancer treatment. PDT relies on the photochemical reactions between excitation light and photosensitizers (PS) to convert molecular oxygen into damaging reactive oxygen species (ROS). Several PS are clinically approved for the treatment of cancers, including porfimer sodium, which is used to treat cervical, endobronchial, esophageal, lung, bladder, gastric, and brain tumors [87,88]. PDT treatment kills cancers by generating ROS that kill tumor cells directly; damage tumor vasculature; and/or activate the immune system to recognize and kills tumor cells [88,89]. Although PDT has been extensively used in clinical settings, patients often suffer from skin photosensitivity. Moreover, utilization of PDT in the treatment of bulky solid tumors is subject to several limitations, including: (i) uneven and low PS accumulation in tumor tissue after systemic administration; (ii) tumor hypoxia that limits the availability of oxygen; and (iii) light penetration within the tumor mass. Therefore, the encapsulation of PS in NDDS has been used increase payloads; avoid photosensitivity by increasing targeting selectivity and reducing unspecific PS accumulation; and to perform combination therapy (e.g., chemo-PDT and photothermal therapy-PDT) [56,88,90].

The use of PNPs as delivery vehicles or PS is in its early stages. For example, the potent hydrophobic PS ZnF16Pc, was loaded into ferritin which was modified to externally-display a single-chain variable fragment (scFv) that binds the fibroblast-activation protein (FAP) overexpressed on CAFs [31]. By targeting PDT to CAFs, the density and stiffness of a tumor’s ECM can be reduced (Figure 3F). ZnF16Pc was encapsulated into scFv–ferritin through a pH-mediated disassembly-reassembly method. This encapsulation method led to a 40 wt.% loading with minimal premature leakage due to the bulky nature and hydrophobicity of ZnF16Pc. ZnF16Pc loaded-scFv ferritin was shown to be stable in solution, with no precipitation observed after storage in PBS for 1 week (Figure 3G) [31,71,91]. Upon irradiation at 671 nm, the PS-loaded ferritin generated ROS in a time-dependent manner (Figure 3H). The presence of scFv promoted a higher ferritin retention in the in vivo tumor section of primary 4T1, LL/2, and LL2 liver metastases. Then, an in vivo study was conducted using bilateral subcutaneous 4T1 tumor-bearing mice model and PDT at a confluence rate of 300mW/cm^2^ for 15 min (Figure 3I). Compared to an unirradiated tumor, the tumor that received PDT showed a reduction in CAFs and collagen levels (observed using Mason’s trichrome staining) (Figure 3J). The decrease of collagen further reduced the density of ECM. Following this PDT treatment, 50 nm quantum dots were able to effectively accumulate and diffuse through the tumor tissue (Figure 3K). This study showed that PS-loaded ferritin could successfully target and mediate PDT to disrupt the TME, improving the subsequent delivery of NDDS and were reported can improve enhanced the infiltration of CD8+ T cells in tumors [92].

### 4.3. Delivery of Biotherapeutics

The clinical translations of biotherapeutics (e.g., genes and proteins) have been hampered by their low stability in blood, potential immunogenicity, poor cell-penetrating properties, and intracellular degradation. Accordingly, an ideal NDDS must protect a biotherapeutic en route to its site-of action (e.g., tumor), enhance its cell-targeting and uptake, and enable it to avoid lysosomal degradation by facilitating its endosomal escape (to the cytosol). By taking advantage of its natural capacity to encapsulate nucleic acids, the MS2 bacteriophage has been widely utilized for their delivery in gene therapy [20,80]. However, other non-viral PNPs have been recently repurposed into vehicles for gene delivery. For example, Guan et al. utilized Hsp for siRNA delivery [37]. Hsp was genetically fused with an arginine-rich peptide (R9) at its C-terminus, resulting in a positively charged exterior surface that facilitated the electrostatic binding of negatively charged siRNA. Using a green fluorescent protein (GFP)-specified siRNA as a model system, Hsp–R9 was able to form a Hsp–R9/siRNA complex at a 1:80 molar ratio (confirmed by gel retardation assay). This Hsp–R9/siRNA complex showed good stability (no siRNA release seen after 4 h in cell culture medium) and protected the siRNA from RNAse degradation. Hsp–R9/siRNA was internalized by HeLa cervical cancer cells via the clathrin-mediated pathway and was able to escape from lysosomes. When compared to free siRNA, the Hsp–R9/siRNA complex showed greater transfection (0.3% verus 35.7%, respectively) and also downregulated GFP expression in HeLa-enhanced GFP cells four times more effectively (determined by measuring changes in fluorescence intensity). This study highlights the potential of Hsp–R9 (and other non-viral PNPs) for gene delivery; however, further investigations are warranted to confirm their feasibility in vivo.

**Figure 4:**
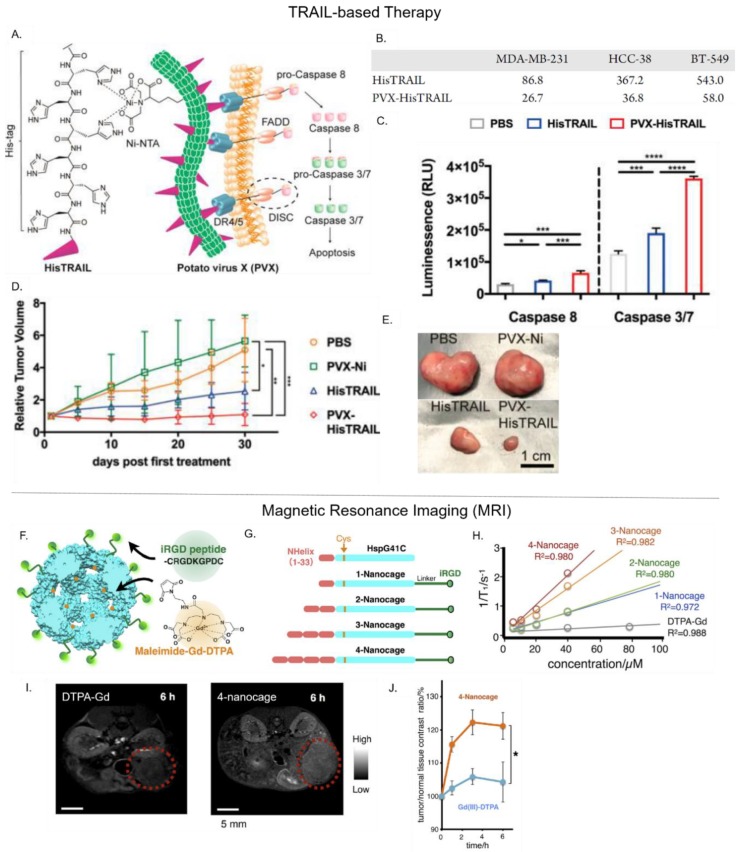
Examples of PNP-mediated delivery of biotherapeutics and imaging agents. (**Upper panel**) TRAIL-based therapy: PVX-mediated delivery of the anti-cancer biotherapeutic HisTRAIL. (**A**) Schematic showing PVX–HisTRAIL-induced apoptosis via caspase dependent pathway. (**B**) IC_50_ of free His-TRAIL and PVX–HisTRAIL against various cancer cell types (**C**). PVX–HisTRAIL activated caspase 8 and caspase 3/7. (**D**,**E**) In comparison to free HisTRAIL, PVX–HisTRAIL significantly regressed tumor growth in tumor-bearing mice after 30 days. Adapted from [24], with permission from American Chemical Society, 2019. (**Lower Panel**) Magnetic resonance imaging (MRI): Hsp-mediated delivery of the MRI contrast agent Gd–DTPA. (**F**) Design of Hsp variants for Gd–DTPA delivery. (**G**) Hsp was modified with a C-terminal iRGD to target pancreatic cancer cells. To generate variants of different sizes, hydrophobic peptides repeats were attached at the N-terminus of Hsp, resulting in: 1-nanocage, 2-nanocage, 3-nanocage, and 4-nanocage. (**H**) Relaxivity values (at 1.5 Tesla) for each iRGD–Hsp variant of free Gd–DTPA, in vitro. (**I**,**J**) In vivo MRI images (9.4 T) in transgenic mice bearing pancreatic tumors 6h after IV injection. The ‘4-nanocage’ variant showed a significantly higher tumor-to-normal MRI contrast ratio than free Gd–DTPA. Adapted from [38], with permission from Elsevier, 2018.

Another potential biotherapeutic for cancer treatment is the tumor necrosis factor (TNF)-related apoptosis-inducing ligand (TRAIL). TRAIL is a cytokine that induces cellular apoptosis by activating caspase-dependent pathways through its interactions with Death Receptors 4 and 5 (DR 4/5). In clinical trials, the soluble form of TRAIL had poor efficacy and high instability. Le et al. developed the PVX PNP to display a multivalent form of TRAIL that mimics its natural membrane-bound state (Figure 4A) [24]. To achieve this, Ni-NTA was conjugated to PVX, which facilitated the subsequent attachment of His-tagged TRAIL (HisTRAIL) in a trimeric form. This approach immobilized TRIAL molecules on the PVX surface in an orientation that exposed its C-terminus, mimicking TRAIL’s native state. The cytotoxic effect of PVX–HisTRAIL on triple negative breast cancer cells was found to be three to five fold higher than free HisTRAIL alone (Figure 4B). It also induced apoptotic caspase activation more effectively (Figure 4C). Furthermore, 30 days after intratumoral injection into tumor-bearing mice, PVX–HisTRAIL was more effective at slowing tumor growth (1.09-fold increase in original tumor size) than free HisTRAIL (2.53-fold increase in original tumor size) (Figure 4D,E). While these results show the clinical potential of PVX–HisTRAIL, further comparative studies must be performed against free TRAIL (e.g., physiological stability and pharmacokinetic profile). 

### 4.4. Delivery of Magnetic Resonance Imaging (MRI) Contrast Agents

MRI is a versatile and noninvasive radiological imaging technique that provides anatomical, physiological, and even molecular information about a physiological system. MR contrast images are produced by measuring the relaxation mechanism of hydrogen protons across the body. Relaxation is referred to the process whereby protons re-align with a magnetic field following their excitation with radiofrequency pulses [93]. To distinguish between MR signals from diseased tissue and surrounding healthy tissue, the contrast in the diseased tissue must be enhanced using contrast agents that have high relaxivity (r1 = 1/T1 or r2 = 1/T2). A common MRI contrast agent is the paramagnetic gadolinium (Gd(III)) ion, which boosts MR image contrast by improving the signal intensity associated with T1-weighted images [94,95]. However, Gd(III) is toxic and must be complexed with a chelating agent [94]. Moreover, clinically-available Gd(III)-based contrast agents (e.g., Gd–DTPA) have low relaxivity (4–5 mM^−1^s^−1^ at 60mHz 25 °C) and are rapidly cleared from the body [96]. Therefore, an ideal NDDS for MRI should have high relaxivity, low toxicity, and the capacity to accumulate within diseased tissue (e.g., tumors).

Recently, Kawano et al. engineered the small heat shock protein 16.5 (Hsp 16.5) PNP into a targeted MRI contrast agent for the detection of pancreatic cancer [38]. Herein, a glycine residue in Hsp 16′s inner cavity was substituted with a cysteine to permit the thiol-maleimide conjugation of Gd–DTPA. This action led to the attachment of one Gd–DTPA per Hsp 16.5 subunit—as quantified by chromatographic methods and mass spectrometry. To selectively target the neurophilin-1 receptor overexpressed on pancreatic cells, the exposed C-terminus of Hsp 16.5 was genetically modified to display iRGD (Figure 4F). Additionally, to evaluate whether the size of Gd–DTPA-loaded iRGD-Hsp 16.5 could affect its overall relaxivity, its N-terminus was modified with varying repeats of a hydrophobic peptide domain (Figure 4G). The variant with four hydrophobic peptide repeats (4-nanocage), displayed 3.5-fold higher relaxivity than a variant with a single hydrophobic peptide, and nine-fold more relaxivity than free Gd–DTPA (Figure 4H). Thus, relaxivity of the Gd–DTPA cargo was dependent upon the PNP carrier’s size, with larger PNPs likely to have slower molecular tumbling rates that result in faster T1 relaxation rates. Furthermore, the cellular uptake of iRGD-displaying Hsp 16.5 by neurophilin-1 positive AsPC-1 pancreatic cells was significantly higher than control cell lines, validating iRGD-Hsp 16.5 capacity to target cancer cells. Finally, in MRIs of tumor-bearing mice, the 4-nanocage efficiently accumulated within tumor periphery 6 h after intravenous administration and increased the tumor-to-normal (T/N)) contrast ratio up to 120%, whereas free Gd–DTPA enhanced T/N by ~105% (Figure 4I,J).

### 4.5. Delivery of Near Infrared Fluorescence (NIRF) Probes

NIRF imaging is a non-invasive technique that measures the fluorescence intensity of fluorophores after excitation with near-infrared (NIR) light (λ 650–900 nm). NIRF imaging provides high spatial resolution and sensitivity and is less harmful than other imaging modalities (e.g., radioimaging) [97]. Only two NIR fluorophores have been approved by the FDA, indocyanine green and methylene blue, while other NIR fluorophores are widely employed in pre-clinical research [98]. In recent years, nanoparticle-based NIRF probes (e.g., quantum dots and upconversion nanoparticles) have been developed to overcome the drawbacks of NIRF dyes including their low photostability, hydrophobicity, and non-specific targeting [97]. In addition, these nanoparticle-based NIRF probes provide the opportunity for multimodal imaging applications.

TMV has been engineered into a PNP-based nanoprobe for MRI/NIRF bimodal imaging [18]. TMV was used to simultaneously deliver the MRI contrast agent dysprosium (Dy^3+^) and the NIR fluorophore Cy7.5. Dy^3+^ is a lanthanide ion with high T2-weighted MR signal intensity that makes it an ideal contrast agent in ultra-high field MRI applications. Both Dy^3+^ and Cy7.5 were conjugated to TMV, resulting in the attachment of ~380 Cy7.5 molecules and ~980 chelated Dy^3+^ ions per TMV particle. The multimodal TMV’s were modified to display either PEG (passive delivery) or the DGEA peptide, which binds integrins overexpressed on prostate cancer cells (active targeting). In comparison to conventional lanthanide-based NaDyF_4_ nanoparticles, Dy–Cy7.5–TMV–PEG had higher *r2* values at 9.4 T (399 versus 101 mM^−1^s^−1^). In a cell model of prostate cancer, actively-targeted Dy–Cy7.5–TMV-DGEA demonstrated about three-fold higher cellular uptake than the passively targeted Dy–Cy7.5–TMV–PEG. Similarly, ex vivo NIRF imaging of mice models, showed that Dy–Cy7.5–TMV-DGEA accumulated inside tumors about two times more effectively than Dy–Cy7.5–TMV–PEG. For in vivo T2-mapping MRI at 9.4 T, Dy–Cy7.5–TMV-DGEA showed a 40% reduction of T2 (which lead to higher value of r2), while Dy–Cy7.5–TMV–PEG showed an only 14% reduction. The outcomes of this research indicate that PNPs can be used for targeted bimodal cancer imaging, allowing for their potential applications in cancer diagnostics, prognostics, and patient stratification.

### 4.6. Delivery of Positron Emission Tomography (PET) Tracers

Positron emission tomography (PET) is an imaging process that provides functional images to diagnose abnormalities at a molecular level [99,100,101]. PET captures diagnostic images using the highly specific radiopharmaceutical activity of its contrast agent [102,103]. This imaging process has a limitless penetration depth and a higher sensitivity than other imaging agents. PET is a very helpful tool for early diagnosis and monitoring disease progression. Although PET is a FDA-approved clinical molecular imaging technique, it does have some drawbacks [104]. PET has low spatial resolution, short acquisition time, and its radioisotopes have a short half-life. In addition, PET can expose patients to large doses of radiation. Therefore, the use of PNPs and some of their multimodal variants can address these issues.

Wang et al. developed ultra-small ^64^Copper sulfide-ferritin nanocages (^64^CuS–Fn) as novel cancer nanotheranostics [33]. ^64^CuS nanoparticles were formed inside the cavity of ferritin (Fn) nanocages using a biomineralization-inspired method. First, ^64^Cu was loaded within ferritin by exploiting the presence of open surface pores and its natural affinity towards bivalent metals (i.e., Fe^2+^). Next, Na_2_S was added into solution to form ^64^CuS–Fn. Due to the presence of the ferritin protein shell, this biomineralization method was used to control the size of ^64^CuS nanoparticle formation, while also enhancing its solubility and monodispersity. ^64^CuS–Fn showed good colloidal stability and minimal copper leakage after 24h incubation in sodium acetate buffer. Eight hours after IV administration in U87 MG subcutaneous tumor-bearing mice, ^64^CuS–Fn doubled the accumulation of ^64^Cu in the tumor area and produced a higher tumor-to-normal contrast PET signal, compared to free ^64^Cu. This approach harnessed the unique properties of PNPs to produce an optically-active ^64^CuS particle with good stability, biocompatibility, and passive targeting. The ferritin shell also provides a scaffold for the attachment of functional cancer-targeting moieties to the encapsulated CuS nanoparticle, which could improve this system’s PET imaging accuracy. Furthermore, CuS nanoparticles have the ability to absorb and convert light into heat, and therefore, this system could be readily applied in photothermal therapy.

### 4.7. Use as Ultrasound Contrast Agents

Ultrasonography (US) is a non-invasive and cost-effective technology that allows anatomical imaging in real-time [105]. US works by transmitting high frequency ultrasound pulses (1–20 mHz) into tissue. The reflection of these pulses off anatomical structures creates echoes that can be detected and then depicted as points of variable brightness (brightness (B)-mode), allowing any changes in tissue anatomy to be observed. Contrast-enhanced ultrasound (CEUS) has been used in the clinic to improve the detection of lesions in breast, liver, kidney, and ovarian cancer. To obtain images suitable for patient diagnosis/prognosis, CEUS must be combined with effective ultrasound contrast agents (UCAs). One class of UCAs are micro or nano-bubbles, which work by resonating ultrasound beams that result in reflection signals greater than those observed in normal tissues [105]. The first FDA-approved UCAs were the albumin-coated microbubbles Albunex^®^ and OptisonTM (GE Healthcare) [106].

Recently, Shapiro et al. described protein-based gas vesicles (GVs) found inside *Anabaena flos-aquae* (Ana GV) and Halobacterium NRC-1 (Halo GV). [39,107]. These GVs are unique rod-shaped PNPs with dimensions between 45–200 nm (width) and 100–800 nm (length) and protein shell thicknesses of ~2 nm [107]. While GVs naturally exclude water from their internal cavities, their protein shells are permeable to gas, thus allowing surrounding gases to diffuse into them. Accordingly, the potential of GVs to act as UCAs has been investigated both in vitro (using a gel phantom model) and in vivo (in immunodeficient mice—via IV or subcutaneous injection). In both studies, the GVs provided clear and robust contrast in ultrasound images, even at nanomolar concentrations [107]. However, before being potentially incorporated into existing clinical US, the safety, biodistribution, and pharmacokinetics of these PNP GVs must be evaluated.

## 5. Conclusions and Future Perspectives

To date, a diverse range of PNPs have been specifically developed as NDDSs for diagnostic and/or therapeutic applications. While showing efficacy in pre-clinical models, engineered PNPs are overly complicated, bringing into question their real-world clinical feasibility. For this reason, we suggest that researchers engineering PNPs adopt a highly rational and standardized approach. For instance, by implementing the MIRIBEL (minimum information reported in bio–nano experimental literature) reporting system that was proposed by the Caruso group to help standardize nanomedicine data and improve its quality and reproducibility [108]. In addition, future efforts to engineer PNPs for nanomedicine should focus on enhancing pharmacokinetics, reducing immunogenicity, and promoting endo/lysosomal escape. Furthermore, rather than using DOX as a drug model to evaluate PNPs, we suggest prioritizing the encapsulation of newer and more promising therapeutics that may have failed initial clinical trials. Finally, in order for PNPs to move from the proof-of-concept stage to feasible clinical applications, they must be developed to effectively leverage the physiological environment of the human body and the pathology of cancer; i.e., taking a “disease-first approach” instead of a “formulation-first approach” [2,108].

## Figures and Tables

**Table 1:** Example of protein-based nanoparticles (PNPs) and their application in cancer therapeutics and imaging agent delivery (in this review).

Type	PNPs	Shape	Size (nm)	Half-Life	Application	Cargo	Functionalization	Ref.
Virus-like Particle (VLP)	Tobacco mosaic virus (TMV)	Cylindrical	300 × 18	30 min	Gene delivery	siRNA	TAT	[15]
					-	-	RGD—PEG spacer	[16] *
					-	-	Serum albumin—PEG	[17] *
					MRI ^1^-NIRF ^2^ imaging	Dy^3+^, Cy7.5	PEG-DGEA targeting peptide	[18] *
	MS2 bacteriophage	Icosahedral	26	N/A	Gene delivery	siRNA	SP94 targeting peptide H5YGW fusogenic peptide	[19]
					Gene delivery	siRNA	TAT	[20] *
	Cowpea mosaic virus (CPMV)	Pseudo-icosahedral	30–34	4–7 min	NIRF imaging	Alexa Fluor	PEG-E7p72 targeting peptide	[21]
					Chemotherapy	Mitoxantrone	-	[22]
	Cowpea chlorotic mottle virus (CCMV)	Icosahedral	28	N/A	Gene delivery	siRNA	M-lycotoxin L17E (penetrating peptide)	[23]
	Potato virus X (PVX)	Filamentous	515 × 13	12.5 min	Protein delivery	TRAIL ^3^	-	[24] *
	Simian Virus 40 (SV40)	Icosahedral	20–40	< than 5 min	NIRF Imaging	Ag_2_S-QD	PEG	[25] *
Non-Virus Like Particle (NVP)	Ferritin	Octahedral	12	1.1 h	Chemotherapy	DOX ^4^	Serum albumin coating	[26] *
					Hypoxia targeting therapy	HIF ^5^-1α inhibitor (Acriflavine)	PEG	[27] *
					Chemotherapy	Mitoxantrone	MMP-cleavable PASE	[28,29] *
					Chemotherapy	Fe (II)-DOX	Nicked ferritin	[30]
					PDT ^6^	ZnF16PC	FAP-scFv	[31] *
					-	-	X-TEN + affibody	[32] *
					PET ^7^	^64^CuS	-	[33] *
	Encapsulin	Icosahedral	20–40	N/A	Chemotherapy	Aldox	SP94-targeting peptide	[34]
	P22	Icosahedral	60	N/A	-	-	CD47-self peptide	[35]
	Heat shock protein (Hsp)	Octahedral	24	N/A	Chemotherapy	OSU030312	iRGD	[36]
					Gene delivery	siRNA	-	[37]
					MRI	Gd–DTPA	iRGD	[38] *
	Gas Vesicles	Tip-conned cylindrical	45 × 250	N/A	Ultrasound	Air	CD47, R8	[39]

Asterisk (*) indicated that the study was conducted in vivo. ^1^ MRI: magnetic resonance imaging, ^2^ NIRF = near infrared fluorescence, ^3^ TRAIL = tumor necrosis factor (TNF)-related apoptosis-inducing ligand, ^4^ DOX=doxorubicin, ^5^ HIF = hypoxia-inducible factor, ^6^ PDT = photodynamic therapy, ^7^ PET = positron emission tomography.

**Table 2:** Engineering strategies used to maximize PNP delivery in vivo.

Purpose	Engineering Strategy	PNP Example in This Review
Prolong circulation half-life	PEGylation	TMV [16], SV40 [25]
	Albumin coating	TMV [17,58], Ferritin [26]
	CD47 “self-peptide”	P22 [35]
	Blood-circulating peptide-1 (BCP-1)	Ferritin [61]
	PAS/PASElation	Ferritin [28,29]
	X-TEN	Ferritin [32]
Increase tumor accumulation	Tumor vasculature disruption (PDT)	Ferritin [71]
	Elongated PNPs	TMV [69], PVX [82]
Improve tumor penetration and diffusion	Receptor-mediated transcytosis	Ferritin [27]
	Elongated/High aspect ratio	TMV [69], PVX [82]
	ECM degradation (via PDT)	Ferritin [31]
Enhance cellular uptake	Cell-targeting peptide (with PEG spacer)	TMV [16]
	Cleavable-stealth coating	Ferritin [28]
Mediate endo/lysosomal escape	Cell-penetrating peptide	MS2 [20], TMV [15], CCMV [23]
	Fusogenic peptide	MS2 [19]

## References

[1] Hartshorn C.M., Bradbury M.S., Lanza G.M., Nel A.E., Rao J., Wang A.Z., Wiesner U.B., Yang L., Grodzinski P. (2018). Nanotechnology Strategies To Advance Outcomes in Clinical Cancer Care. ACS Nano.

[2] Björnmalm M., Thurecht K.J., Michael M., Scott A.M., Caruso F. (2017). Bridging Bio–Nano Science and Cancer Nanomedicine. ACS Nano.

[3] Shi J., Kantoff P.W., Wooster R., Farokhzad O.C. (2017). Cancer nanomedicine: Progress, challenges and opportunities. Nat. Rev. Cancer.

[4] Chapman S., Dobrovolskaia M., Farahani K., Goodwin A., Joshi A., Lee H., Meade T., Pomper M., Ptak K., Rao J. (2013). Nanoparticles for cancer imaging: The good, the bad, and the promise. Nano Today.

[5] Hare J.I., Lammers T., Ashford M.B., Puri S., Storm G., Barry S.T. (2017). Challenges and strategies in anti-cancer nanomedicine development: An industry perspective. Adv. Drug Deliv. Rev..

[6] Bobo D., Robinson K.J., Islam J., Thurecht K.J., Corrie S.R. (2016). Nanoparticle-Based Medicines: A Review of FDA-Approved Materials and Clinical Trials to Date. Pharm. Res..

[7] Rosenblum D., Joshi N., Tao W., Karp J.M., Peer D. (2018). Progress and challenges towards targeted delivery of cancer therapeutics. Nat. Commun..

[8] Wicki A., Witzigmann D., Balasubramanian V., Huwyler J. (2015). Nanomedicine in cancer therapy: Challenges, opportunities, and clinical applications. J. Control Release.

[9] Wilhelm S., Tavares A.J., Dai Q., Ohta S., Audet J., Dvorak H.F., Chan W.C.W. (2016). Analysis of nanoparticle delivery to tumours. Nat. Rev. Mater..

[10] Diaz D., Care A., Sunna A. (2018). Bioengineering Strategies for Protein-Based Nanoparticles. Genes.

[11] Lee E.J., Lee N.K., Kim I.-S. (2016). Bioengineered protein-based nanocage for drug delivery. Adv. Drug Deliv. Rev..

[12] Molino N.M., Wang S.-W. (2014). Caged protein nanoparticles for drug delivery. Curr. Opin. Biotechnol..

[13] Schoonen L., van Hest J.C.M. (2014). Functionalization of protein-based nanocages for drug delivery applications. Nanoscale.

[14] Ferrer-Miralles N., Rodríguez-Carmona E., Corchero J.L., García-Fruitós E., Vázquez E., Villaverde A. (2015). Engineering protein self-assembling in protein-based nanomedicines for drug delivery and gene therapy. Crit. Rev. Biotechnol..

[15] Tian Y., Zhou M., Shi H., Gao S., Xie G., Zhu M., Wu M., Chen J., Niu Z. (2018). Integration of Cell-Penetrating Peptides with Rod-like Bionanoparticles: Virus-Inspired Gene-Silencing Technology. Nano Lett..

[16] Pitek A.S., Wen A.M., Shukla S., Steinmetz N.F. (2016). The Protein Corona of Plant Virus Nanoparticles Influences their Dispersion Properties, Cellular Interactions, and In Vivo Fates. Small.

[17] Pitek A.S., Jameson S.A., Veliz F.A., Shukla S., Steinmetz N.F. (2016). Serum albumin ‘camouflage’ of plant virus based nanoparticles prevents their antibody recognition and enhances pharmacokinetics. Biomaterials.

[18] Hu H., Zhang Y., Shukla S., Gu Y., Yu X., Steinmetz N.F. (2017). Dysprosium-Modified Tobacco Mosaic Virus Nanoparticles for Ultra-High-Field Magnetic Resonance and Near-Infrared Fluorescence Imaging of Prostate Cancer. ACS Nano.

[19] Ashley C.E., Carnes E.C., Phillips G.K., Durfee P.N., Buley M.D., Lino C.A., Padilla D.P., Phillips B., Carter M.B., Willman C.L. (2011). Cell-specific delivery of diverse cargos by bacteriophage MS2 virus-like particles. ACS Nano.

[20] Wang G., Jia T., Xu X., Chang L., Zhang R., Fu Y., Li Y., Yang X., Zhang K., Lin G. (2016). Novel miR-122 delivery system based on MS2 virus like particle surface displaying cell-penetrating peptide TAT for hepatocellular carcinoma. Oncotarget.

[21] Cho C.-F., Yu L., Nsiama T.K., Kadam A.N., Raturi A., Shukla S., Amadei G.A., Steinmetz N.F., Luyt L.G., Lewis J.D. (2017). Viral nanoparticles decorated with novel EGFL7 ligands enable intravital imaging of tumor neovasculature. Nanoscale.

[22] Lam P., Lin R.D., Steinmetz N.F. (2018). Delivery of mitoxantrone using a plant virus-based nanoparticle for the treatment of glioblastomas. J. Mater. Chem. B.

[23] Lam P., Steinmetz N.F. (2019). Delivery of siRNA therapeutics using cowpea chlorotic mottle virus-like particles. Biomater. Sci..

[24] Le D.H.T., Commandeur U., Steinmetz N.F. (2019). Presentation and Delivery of Tumor Necrosis Factor-Related Apoptosis-Inducing Ligand via Elongated Plant Viral Nanoparticle Enhances Antitumor Efficacy. ACS Nano.

[25] Li C., Li F., Zhang Y., Zhang W., Zhang X.-E., Wang Q. (2015). Real-Time Monitoring Surface Chemistry-Dependent In Vivo Behaviors of Protein Nanocages via Encapsulating an NIR-II Ag2S Quantum Dot. ACS Nano.

[26] Wang C., Zhang C., Li Z., Yin S., Wang Q., Guo F., Zhang Y., Yu R., Liu Y., Su Z. (2018). Extending Half Life of H-Ferritin Nanoparticle by Fusing Albumin Binding Domain for Doxorubicin Encapsulation. Biomacromolecules.

[27] Huang X., Zhuang J., Chung S.W., Huang B., Halpert G., Negron K., Sun X., Yang J., Oh Y., Hwang P.M. (2019). Hypoxia-tropic Protein Nanocages for Modulation of Tumor- and Chemotherapy-Associated Hypoxia. ACS Nano.

[28] Falvo E., Malagrino F., Arcovito A., Fazi F., Colotti G., Tremante E., Di Micco P., Braca A., Opri R., Giuffre A. (2018). The presence of glutamate residues on the PAS sequence of the stimuli-sensitive nano-ferritin improves in vivo biodistribution and mitoxantrone encapsulation homogeneity. J. Control Release.

[29] Fracasso G., Falvo E., Colotti G., Fazi F., Ingegnere T., Amalfitano A., Doglietto G.B., Alfieri S., Boffi A., Morea V. (2016). Selective delivery of doxorubicin by novel stimuli-sensitive nano-ferritins overcomes tumor refractoriness. J. Control Release.

[30] Ahn B., Lee S.-G., Yoon H.R., Lee J.M., Oh H.J., Kim H.M., Jung Y. (2018). Four-fold Channel-Nicked Human Ferritin Nanocages for Active Drug Loading and pH-Responsive Drug Release. Angew. Chem. Int. Ed..

[31] Li L., Zhou S., Lv N., Zhen Z., Liu T., Gao S., Xie J., Ma Q. (2018). Photosensitizer-Encapsulated Ferritins Mediate Photodynamic Therapy against Cancer-Associated Fibroblasts and Improve Tumor Accumulation of Nanoparticles. Mol. Pharm..

[32] Lee N.K., Lee E.J., Kim S., Nam G.-H., Kih M., Hong Y., Jeong C., Yang Y., Byun Y., Kim I.-S. (2017). Ferritin nanocage with intrinsically disordered proteins and affibody: A platform for tumor targeting with extended pharmacokinetics. J. Control Release.

[33] Wang Z., Huang P., Jacobson O., Wang Z., Liu Y., Lin L., Lin J., Lu N., Zhang H., Tian R. (2016). Biomineralization-Inspired Synthesis of Copper Sulfide-Ferritin Nanocages as Cancer Theranostics. ACS Nano.

[34] Moon H., Lee J., Min J., Kang S. (2014). Developing genetically engineered encapsulin protein cage nanoparticles as a targeted delivery nanoplatform. Biomacromolecules.

[35] Schwarz B., Madden P., Avera J., Gordon B., Larson K., Miettinen H.M., Uchida M., LaFrance B., Basu G., Rynda-Apple A. (2015). Symmetry Controlled, Genetic Presentation of Bioactive Proteins on the P22 Virus-like Particle Using an External Decoration Protein. ACS Nano.

[36] Murata M., Narahara S., Kawano T., Hamano N., Piao J.S., Kang J.H., Ohuchida K., Murakami T., Hashizume M. (2015). Design and Function of Engineered Protein Nanocages as a Drug Delivery System for Targeting Pancreatic Cancer Cells via Neuropilin-1. Mol. Pharm..

[37] Guan X., Chang Y., Sun J., Song J., Xie Y. (2018). Engineered Hsp Protein Nanocages for siRNA Delivery. Macromol. Biosci..

[38] Kawano T., Murata M., Kang J.-H., Piao J.S., Narahara S., Hyodo F., Hamano N., Guo J., Oguri S., Ohuchida K. (2018). Ultrasensitive MRI detection of spontaneous pancreatic tumors with nanocage-based targeted contrast agent. Biomaterials.

[39] Lakshmanan A., Farhadi A., Nety S.P., Lee-Gosselin A., Bourdeau R.W., Maresca D., Shapiro M.G. (2016). Molecular Engineering of Acoustic Protein Nanostructures. ACS Nano.

[40] Ginn S.L., Amaya A.K., Alexander I.E., Edelstein M., Abedi M.R. (2018). Gene therapy clinical trials worldwide to 2017: An update. J. Gene Med..

[41] Rohovie M.J., Nagasawa M., Swartz J.R. (2017). Virus-like particles: Next-generation nanoparticles for targeted therapeutic delivery. Bioeng. Transl. Med..

[42] Ungerechts G., Bossow S., Leuchs B., Holm P.S., Rommelaere J., Coffey M., Coffin R., Bell J., Nettelbeck D.M. (2016). Moving oncolytic viruses into the clinic: Clinical-grade production, purification, and characterization of diverse oncolytic viruses. Mol. Ther. Methods Clin. Dev..

[43] Wang J.W., Roden R.B.S. (2013). Virus-like particles for the prevention of human papillomavirus-associated malignancies. Expert Rev. Vaccines.

[44] Sun Q., Sun X., Ma X., Zhou Z., Jin E., Zhang B., Shen Y., Van Kirk E.A., Murdoch W.J., Lott J.R. (2014). Integration of Nanoassembly Functions for an Effective Delivery Cascade for Cancer Drugs. Adv. Mater..

[45] Bhaskar S., Lim S. (2017). Engineering protein nanocages as carriers for biomedical applications. Npg Asia Mater..

[46] Singh P., Prasuhn D., Yeh R.M., Destito G., Rae C.S., Osborn K., Finn M.G., Manchester M. (2007). Bio-distribution, toxicity and pathology of cowpea mosaic virus nanoparticles in vivo. J. Control Release.

[47] Bruckman M.A., Randolph L.N., VanMeter A., Hern S., Shoffstall A.J., Taurog R.E., Steinmetz N.F. (2014). Biodistribution, pharmacokinetics, and blood compatibility of native and PEGylated tobacco mosaic virus nano-rods and -spheres in mice. Virology.

[48] Shukla S., Wen A.M., Ayat N.R., Commandeur U., Gopalkrishnan R., Broome A.-M., Lozada K.W., Keri R.A., Steinmetz N.F. (2014). Biodistribution and clearance of a filamentous plant virus in healthy and tumor-bearing mice. Nanomedicine.

[49] Kaiser C.R., Flenniken M.L., Gillitzer E., Harmsen A.L., Harmsen A.G., Jutila M.A., Douglas T., Young M.J. (2007). Biodistribution studies of protein cage nanoparticles demonstrate broad tissue distribution and rapid clearance in vivo. Int. J. Nanomed..

[50] Nguyen V.H., Lee B.-J. (2017). Protein corona: A new approach for nanomedicine design. Int. J. Nanomed..

[51] Corbo C., Molinaro R., Parodi A., Toledano Furman N.E., Salvatore F., Tasciotti E. (2016). The impact of nanoparticle protein corona on cytotoxicity, immunotoxicity and target drug delivery. Nanomedicine.

[52] Aanei I.L., ElSohly A.M., Farkas M.E., Netirojjanakul C., Regan M., Taylor Murphy S., O’Neil J.P., Seo Y., Francis M.B. (2016). Biodistribution of Antibody-MS2 Viral Capsid Conjugates in Breast Cancer Models. Mol. Pharm..

[53] Fang Y., Xue J., Gao S., Lu A., Yang D., Jiang H., He Y., Shi K. (2017). Cleavable PEGylation: A strategy for overcoming the “PEG dilemma” in efficient drug delivery. Drug Deliv..

[54] Gulati N.M., Stewart P.L., Steinmetz N.F. (2018). Bioinspired Shielding Strategies for Nanoparticle Drug Delivery Applications. Mol. Pharm..

[55] Vaitkuviene A., Kaseta V., Voronovic J., Ramanauskaite G., Biziuleviciene G., Ramanaviciene A., Ramanavicius A. (2013). Evaluation of cytotoxicity of polypyrrole nanoparticles synthesized by oxidative polymerization. J. Hazard. Mater..

[56] Khaliq N.U., Oh K.S., Sandra F.C., Joo Y., Lee J., Byun Y., Kim I.-S., Kwon I.C., Seo J.H., Kim S.Y. (2017). Assembly of polymer micelles through the sol-gel transition for effective cancer therapy. J. Control Release.

[57] Gulati N.M., Pitek A.S., Czapar A.E., Stewart P.L., Steinmetz N.F. (2018). The in vivo fates of plant viral nanoparticles camouflaged using self-proteins: Overcoming immune recognition. J. Mater. Chem. B.

[58] Pitek A.S., Hu H., Shukla S., Steinmetz N.F. (2018). Cancer Theranostic Applications of Albumin-Coated Tobacco Mosaic Virus Nanoparticles. ACS Appl. Mater. Interfaces.

[59] Rodriguez P.L., Harada T., Christian D.A., Pantano D.A., Tsai R.K., Discher D.E. (2013). Minimal “Self” peptides that inhibit phagocytic clearance and enhance delivery of nanoparticles. Science.

[60] Wang Y., Wang Z., Qian Y., Fan L., Yue C., Jia F., Sun J., Hu Z., Wang W. (2019). Synergetic estrogen receptor-targeting liposome nanocarriers with anti-phagocytic properties for enhanced tumor theranostics. J. Mater. Chem. B.

[61] Jin P., Sha R., Zhang Y., Liu L., Bian Y., Qian J., Qian J., Lin J., Ishimwe N., Hu Y. (2019). Blood Circulation-Prolonging Peptides for Engineered Nanoparticles Identified via Phage Display. Nano Lett..

[62] Ayer M., Klok H.-A. (2017). Cell-mediated delivery of synthetic nano- and microparticles. J. Control Release.

[63] Singh B., Mitragotri S. (2019). Harnessing cells to deliver nanoparticle drugs to treat cancer. Biotechnol. Adv..

[64] Falvo E., Tremante E., Arcovito A., Papi M., Elad N., Boffi A., Morea V., Conti G., Toffoli G., Fracasso G. (2016). Improved Doxorubicin Encapsulation and Pharmacokinetics of Ferritin–Fusion Protein Nanocarriers Bearing Proline, Serine, and Alanine Elements. Biomacromolecules.

[65] Chung A.S., Lee J., Ferrara N. (2010). Targeting the tumour vasculature: Insights from physiological angiogenesis. Nat. Rev. Cancer.

[66] Jain R.K., Stylianopoulos T. (2010). Delivering nanomedicine to solid tumors. Nat. Rev. Clin. Oncol..

[67] Chauhan V.P., Jain R.K. (2013). Strategies for advancing cancer nanomedicine. Nat. Mater..

[68] Stylianopoulos T., Jain R.K. (2015). Design considerations for nanotherapeutics in oncology. Nanomedicine.

[69] Shukla S., Eber F.J., Nagarajan A.S., DiFranco N.A., Schmidt N., Wen A.M., Eiben S., Twyman R.M., Wege C., Steinmetz N.F. (2015). The Impact of Aspect Ratio on the Biodistribution and Tumor Homing of Rigid Soft-Matter Nanorods. Adv. Healthc. Mater..

[70] Nakamura Y., Mochida A., Choyke P.L., Kobayashi H. (2016). Nanodrug Delivery: Is the Enhanced Permeability and Retention Effect Sufficient for Curing Cancer?. Bioconjug. Chem..

[71] Zhen Z., Tang W., Chuang Y.J., Todd T., Zhang W., Lin X., Niu G., Liu G., Wang L., Pan Z. (2014). Tumor vasculature targeted photodynamic therapy for enhanced delivery of nanoparticles. ACS Nano.

[72] Valkenburg K.C., de Groot A.E., Pienta K.J. (2018). Targeting the tumour stroma to improve cancer therapy. Nat. Rev. Clin. Oncol..

[73] Dvorak H.F. (1986). Tumors: Wounds That Do Not Heal. N. Engl. J. Med..

[74] Wu H., Wang J., Wang Z., Fisher D.R., Lin Y. (2008). Apoferritin-templated yttrium phosphate nanoparticle conjugates for radioimmunotherapy of cancers. J. Nanosci. Nanotechnol..

[75] Huang X., Chisholm J., Zhuang J., Xiao Y., Duncan G., Chen X., Suk J.S., Hanes J. (2017). Protein nanocages that penetrate airway mucus and tumor tissue. Proc. Natl. Acad. Sci. USA.

[76] Akinc A., Battaglia G. (2013). Exploiting endocytosis for nanomedicines. Cold Spring Harb. Perspect. Biol..

[77] Yameen B., Choi W.I., Vilos C., Swami A., Shi J., Farokhzad O.C. (2014). Insight into nanoparticle cellular uptake and intracellular targeting. J. Control Release.

[78] Pelkmans L., Kartenbeck J., Helenius A. (2001). Caveolar endocytosis of simian virus 40 reveals a new two-step vesicular-transport pathway to the ER. Nat. Cell Biol..

[79] Plummer E.M., Manchester M. (2013). Endocytic Uptake Pathways Utilized by CPMV Nanoparticles. Mol. Pharm..

[80] Chang L., Wang G., Jia T., Zhang L., Li Y., Han Y., Zhang K., Lin G., Zhang R., Li J. (2016). Armored long non-coding RNA MEG3 targeting EGFR based on recombinant MS2 bacteriophage virus-like particles against hepatocellular carcinoma. Oncotarget.

[81] Karagiannis E.D., Urbanska A.M., Sahay G., Pelet J.M., Jhunjhunwala S., Langer R., Anderson D.G. (2013). Rational design of a biomimetic cell penetrating peptide library. ACS Nano.

[82] Shukla S., Ablack A.L., Wen A.M., Lee K.L., Lewis J.D., Steinmetz N.F. (2013). Increased Tumor Homing and Tissue Penetration of the Filamentous Plant Viral Nanoparticle Potato virus X. Mol. Pharm..

[83] Kim M., Rho Y., Jin K.S., Ahn B., Jung S., Kim H., Ree M. (2011). pH-Dependent Structures of Ferritin and Apoferritin in Solution: Disassembly and Reassembly. Biomacromolecules.

[84] Petrova V., Annicchiarico-Petruzzelli M., Melino G., Amelio I. (2018). The hypoxic tumour microenvironment. Oncogenesis.

[85] Zhen Z., Tang W., Chen H., Lin X., Todd T., Wang G., Cowger T., Chen X., Xie J. (2013). RGD-Modified Apoferritin Nanoparticles for Efficient Drug Delivery to Tumors. ACS Nano.

[86] Wang Q., Zhang C., Liu L., Li Z., Guo F., Li X., Luo J., Zhao D., Liu Y., Su Z. (2017). High hydrostatic pressure encapsulation of doxorubicin in ferritin nanocages with enhanced efficiency. J. Biotechnol..

[87] Van Straten D., Mashayekhi V., De Bruijn H.S., Oliveira S., Robinson D.J. (2017). Oncologic Photodynamic Therapy: Basic Principles, Current Clinical Status and Future Directions. Cancers.

[88] Lucky S.S., Soo K.C., Zhang Y. (2015). Nanoparticles in Photodynamic Therapy. Chem. Rev..

[89] Dolmans D.E.J.G.J., Fukumura D., Jain R.K. (2003). Photodynamic therapy for cancer. Nat. Rev. Cancer.

[90] Khaliq N.U., Park D.Y., Lee H.J., Oh K.S., Seo J.H., Kim S.Y., Hwang C.S., Lim T.-H., Yuk S.H. (2018). Pluronic/Heparin Nanoparticles for Chemo-Photodynamic Combination Cancer Therapy through Photoinduced Caspase-3 Activation. ACS Appl. Nano Mater..

[91] Zhen Z., Tang W., Guo C., Chen H., Lin X., Liu G., Fei B., Chen X., Xu B., Xie J. (2013). Ferritin Nanocages To Encapsulate and Deliver Photosensitizers for Efficient Photodynamic Therapy against Cancer. ACS Nano.

[92] Zhen Z., Tang W., Wang M., Zhou S., Wang H., Wu Z., Hao Z., Li Z., Liu L., Xie J. (2017). Protein Nanocage Mediated Fibroblast-Activation Protein Targeted Photoimmunotherapy To Enhance Cytotoxic T Cell Infiltration and Tumor Control. Nano Lett..

[93] Shukla S., Steinmetz N.F. (2015). Virus-based nanomaterials as positron emission tomography and magnetic resonance contrast agents: From technology development to translational medicine. Wiley Interdiscip. Rev. Nanomed. Nanobiotechnol..

[94] Caravan P. (2006). Strategies for increasing the sensitivity of gadolinium based MRI contrast agents. Chem. Soc. Rev..

[95] Lauffer R.B. (1987). Paramagnetic metal complexes as water proton relaxation agents for NMR imaging: Theory and design. Chem. Rev..

[96] Garimella P.D., Datta A., Romanini D.W., Raymond K.N., Francis M.B. (2011). Multivalent, high-relaxivity MRI contrast agents using rigid cysteine-reactive gadolinium complexes. J. Am. Chem. Soc..

[97] He X., Gao J., Gambhir S.S., Cheng Z. (2010). Near-infrared fluorescent nanoprobes for cancer molecular imaging: Status and challenges. Trends Mol. Med..

[98] Singh N., Kumar P., Riaz U. (2019). Applications of near infrared and surface enhanced Raman scattering techniques in tumor imaging: A short review. Spectrochim. Acta A Mol. Biomol. Spectrosc..

[99] Basu S., Alavi A. (2016). PET-Based Personalized Management in Clinical Oncology: An Unavoidable Path for the Foreseeable Future. PET Clin..

[100] Torigian D.A., Kjaer A., Zaidi H., Alavi A. (2016). PET/MR Imaging: Clinical Applications. PET Clin..

[101] Zhou M., Zhang R., Huang M., Lu W., Song S., Melancon M.P., Tian M., Liang D., Li C. (2010). A chelator-free multifunctional [^64^Cu]CuS nanoparticle platform for simultaneous micro-PET/CT imaging and photothermal ablation therapy. J. Am. Chem. Soc..

[102] Aghanejad A., Jalilian A.R., Ardaneh K., Bolourinovin F., Yousefnia H., Samani A.B. (2015). Preparation and Quality Control of (68)Ga-Citrate for PET Applications. Asia Ocean J. Nucl. Med. Biol..

[103] Zeng D., Lee N.S., Liu Y., Zhou D., Dence C.S., Wooley K.L., Katzenellenbogen J.A., Welch M.J. (2012). 64Cu Core-labeled nanoparticles with high specific activity via metal-free click chemistry. ACS Nano.

[104] Hahn M.A., Singh A.K., Sharma P., Brown S.C., Moudgil B.M. (2011). Nanoparticles as contrast agents for in-vivo bioimaging: Current status and future perspectives. Anal. Bioanal. Chem..

[105] Wischhusen J., Padilla F. (2019). Ultrasound Molecular Imaging with Targeted Microbubbles for Cancer Diagnostics: From Bench to Bedside. IRBM.

[106] Sirsi S., Borden M. (2009). Microbubble Compositions, Properties and Biomedical Applications. Bubble Sci. Eng. Technol..

[107] Shapiro M.G., Goodwill P.W., Neogy A., Yin M., Foster F.S., Schaffer D.V., Conolly S.M. (2014). Biogenic gas nanostructures as ultrasonic molecular reporters. Nat. Nanotechnol..

[108] Faria M., Björnmalm M., Thurecht K.J., Kent S.J., Parton R.G., Kavallaris M., Johnston A.P.R., Gooding J.J., Corrie S.R., Boyd B.J. (2018). Minimum information reporting in bio–nano experimental literature. Nat. Nanotechnol..

